# A Metabolome Atlas of Grape Biodiversity

**DOI:** 10.1021/acs.jafc.6c04648

**Published:** 2026-08-25

**Authors:** Panagiotis Arapitsas, Pietro Franceschi, Mar Garcia-Aloy, Urska Vrhovsek, Luca Narduzzi, Domenico Masuero, Marco Stefanini, Paola Bettinelli, Maria Stella Grando, Francesco Emanuelli, Ron Wehrens, Georgios Theodoridis, Fulvio Mattivi

**Affiliations:** Research and Innovation Centre, 4508 Fondazione Edmund Mach, San Michele all’Adige 38098, Italy

**Keywords:** Grape metabolomics, Polyphenols, Terpenes, Chemotaxonomy, Phenotyping, *Vitis*, Metabolome, UMAP

## Abstract

Grapevine (*Vitis* spp.) is one of the world’s most economically, culturally, and biologically important fruit crops, with remarkable diversity spanning thousands of *V. vinifera* cultivars together with rootstocks and wild relatives. Yet grapevine biodiversity has never been systematically explored at the metabolome scale, despite its importance for chemotaxonomy, breeding, and sustainable viticulture. Here, we present the Grape Metabolome (GM) Project, the first systematic liquid chromatography–mass spectrometry (LC–MS) survey of grapevine biodiversity, comprising 462 samples from 126 accessions collected across four vintages. Advanced and well-established chemometric tools resolved the major grape chemotypes together with finer cultivar-related substructure, while annotation-guided interpretation linked this organization to distinctive patterns in anthocyanins, flavonols, aroma-related compounds, and tannin-associated phenolics. This data set provides a reference framework for chemotaxonomy, breeding, authenticity, precision viticulture and enology.

## Graphical abstract

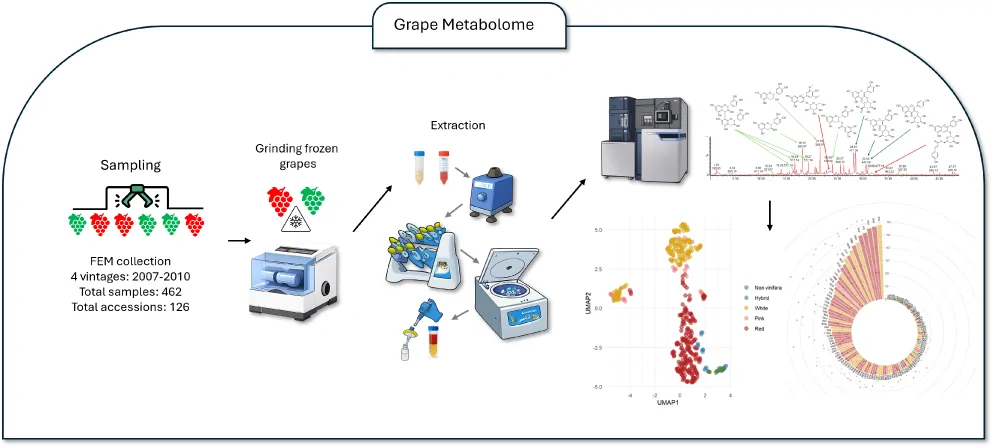

## 1 Introduction

The grapevine (*Vitis* spp.) is a woody perennial liana which has accompanied human societies for millennia and has an ancient, global evolutionary history, providing fruit, wine, and cultural symbolism that remains central to many civilizations. Archeological evidence suggests that grape domestication, cultivation and winemaking began more than 8,000 years ago in the South Caucasus, and since then the grapevine has spread worldwide.^1,2^ Today, grapes represent one of the most widely cultivated and economically important fruit crops, supporting diverse industries from table grape and raisin production to juice and, most significantly, wine. In 2024, the global vineyard surface area was estimated at 7.1 million hectares, and the global trade in wine alone exceeds 35 billion euros annually.^3^ The genus *Vitis* is taxonomically diverse, comprising around 60 species. Among them *Vitis vinifera* L. has been the dominant cultivated species, giving rise to thousands of cultivars adapted to a wide range of terroirs. The *Vitis* International Variety Catalogue (*V*IVC) currently lists approximately 6,000 genetically distinct *V. vinifera* cultivars.^4^ In Europe alone, more than 1,900 varieties are officially registered for cultivation.^5^ This biodiversity underpins the resilience of viticulture, the adaptability of grapevine to climate and biotic pressures, and at the same time, it represents a major challenge for conservation and characterization.

Beyond their agronomic and cultural value, grapes are a major dietary source of phytochemicals. Polyphenols such as anthocyanins, flavonols, stilbenes, flavanols and proanthocyanidins contribute not only to berry physiology and plant defense but also to wine quality, color, astringency, and aging potential.^6^ These compounds have been widely studied for their potential health-promoting properties, including antioxidant, cardioprotective, and anti-inflammatory effects.^7^ However, grapes are a chemically very complex matrix, containing an unknown number of metabolites spanning primary and secondary metabolism and arising from diverse biosynthetic pathways, including the shikimate–phenylpropanoid route, the terpenoid pathway, amino acid and alkaloid metabolism, fatty acid and lipid synthesis, and carbohydrate and organic acid metabolism.^8^ Grapevine has therefore become a model species for research in plant secondary metabolism, stress biology, and food chemistry. Despite this importance, untargeted metabolomics studies systematically exploring grapevine metabolome diversity across its biodiversity remain scarce. Considering the extent of grapevine biodiversity and its global significance, there is a need for comprehensive, metabolome-scale approaches that can capture thousands of features simultaneously, provide reliable annotation, and generate reusable Big-Data resources for the community.

Metabolomics, defined as the comprehensive analysis of small molecules in a biological system, has emerged as a powerful complement to genomics, transcriptomics, and proteomics by capturing the real-time biochemical phenotype.^9,10^ In grape and wine research, metabolomics has already demonstrated its ability to distinguish cultivars,^11–13^ terroirs,^14^ and quality categories,^14,15^ to investigate berry responses to biotic and abiotic stresses,^16,17^ and to monitor wine aging and processing.^18–23^ However, most studies have been restricted in scale, typically addressing a limited number of varieties or compound classes. Previous LC–MS studies on grapevine woody tissues have also explored cultivar-dependent variation in selected phenolic compounds, but these works were focused on canes/wood biomass and on narrower chemical fractions than the broad berry data set presented here.^24,25^ What has been missing is a systematic, large-scale, open-access metabolomics survey of cultivated *Vitis vinifera* biodiversity.

In light of this gap, the aim of this work is to present the Grape Metabolome (GM), through a systematic LC–MS based analysis of grapevine metabolomic biodiversity of 462 samples, representing 126 *Vitis* accessions, over four harvests. The resulting data set is intended to help the wine-science community formulate and test hypotheses and to provide a training resource for computational metabolomics research.

## 2 Materials and Methods

### 2.1 Genetic Material

All the accessions were part of the ampelographic collection of the Fondazione Edmund Mach (international code ITA362). The grapevine panel was selected to represent the six major genetic clusters identified by Emanuelli et al.^20^ based on population structure analyses of simple sequence repeat (SSR) marker data. In that study, at K = 5, cultivated *V. vinifera* ssp. *sativa* accessions are partitioned into three subpopulations: S1, comprising Mediterranean wine and table grapes; S2, including muscat-flavored wine and table grapes; and S3, encompassing wine cultivars from Central Europe. A distinct group corresponds to *V. vinifera* ssp. *sylvestris* (VS), which also includes sativa cultivars showing sylvestris-related ancestry.^21^ The remaining groups include non-vinifera (Rs) and admixed accessions (ADM), characterized by the absence of a predominant cluster membership. The panel also included accessions not analyzed by Emanuelli et al.,^20^ namely specific hybrids between American species and *V. vinifera* (Hybrid F1), along with additional relevant cultivars (UNK). The sampling design was intended to ensure representation of these genetic groups focusing primarily on *V. vinifera*. Assignment to clusters based on the STRUCTURE SSR-membership by Emanuelli et al.^20^ of the selected accessions is reported in Table S1 (column “Structure”), together with the “HYBRID F1” and “UNK” classes.^22^

### 2.2 Sampling and Sample Preparation

All grape samples included in the study were collected from vines cultivated using a standardized system, with Guyot trellising, in vineyards with rows north–south oriented. Healthy grapes were sampled in 2007 (103 accessions), 2008 (120 accessions), 2009 (118 accessions), and 2010 (121 accessions) at a standardized soluble-solids stage of approximately 18 °Brix (Figure 1). Ripening was monitored approximately every 3 days to identify the accessions closest to the target harvest value. For each accession and vintage, approximately 1 kg of berries was collected as a pooled field sample from three predefined vines in the collection. The pool was assembled by collecting small bunch portions from different clusters and from different positions on each vine, in order to better represent within-vine and between-vine variability. A pooled sample of six varieties (Sangiovese, Cabernet Sauvignon, Merlot, Riesling, Chardonnay and Gewürztraminer), covering diverse berry chemotypes, was selected as the Quality Control (QC) sample. After collecting, all samples were directly frozen and stored at −80 °C. Sample homogenization was performed at the end of the 4-year sampling campaign by grinding a representative 200 g aliquot of frozen berries under liquid nitrogen with an IKA A11 homogenizer (IKA, Staufen, Germany). The resulting frozen powder was thoroughly homogenized, stored in 50 mL Falcon tubes at −80 °C, and 1 g of this homogenized powder was subsequently weighed for metabolite extraction (Figure 1).

**Figure 1:**
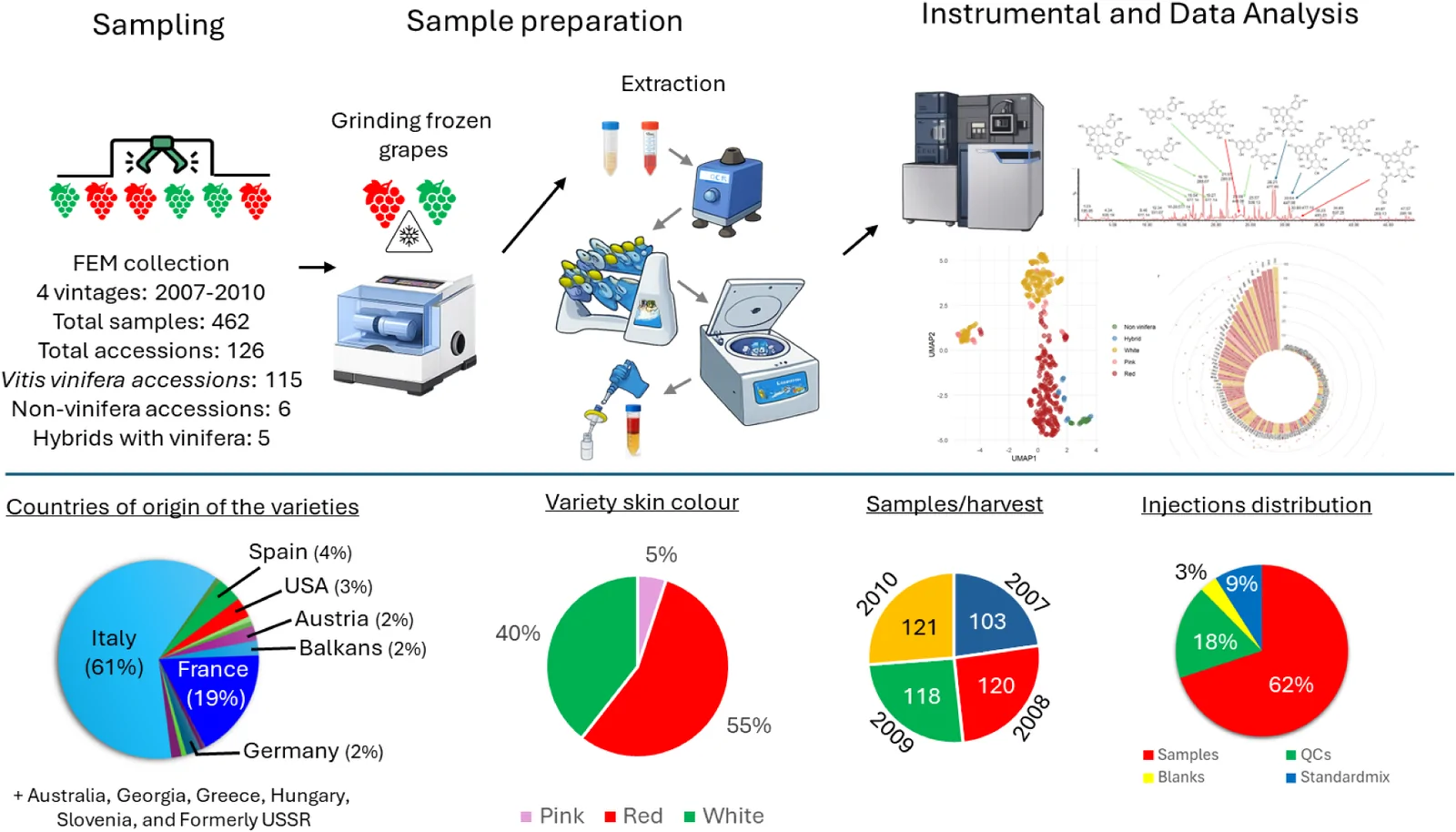
Experimental design and workflow.

The standard mix sample (STDmix) included *trans*-resveratrol, caffeic acid, ellagic acid, catechin, quercetin, quercetin-3-glucoside, cyanidin-3-galactoside, methyl stearate, 4-aminobutyric acid, glutathione and ursolic acid in methanol/water (70/30). For the metabolite extraction the optimized protocol developed by Theodoridis et al. was used.^23^ Briefly, 1 g of each sample powder was weighed into 15 mL amber vials and then 20 μL of the internal standard (IS) (4800 mg/L 4-OH-stilbene, 3000 mg/L gentisic acid, 1200 mg/L indol-propionic acid in methanol/water (1/1 v/v)), 1.2 mL of methanol/water (2:1) and 0.8 mL of chloroform were added. After vortexing for 1 min, each sample was agitated in an orbital shaker (Rotator PTR-60, Grant-Bio, Royston, UK) for 15 min at room temperature and then centrifuged at 1000 × g and 4 °C for 10 min. The upper aqueous methanol phase was collected and filtered through 0.2 μm polytetrafluoroethylene (PTFE) filters (Millipore, Burlington, MA, USA) in 2 mL amber (LC-MS certificated) vials (Agilent Technologies, Santa Clara, CA, USA). All chemicals were purchased by Sigma-Aldrich (Milan, Italy) and LC-MS grade solvents by Honeywell Riedel-de Haën (Seelze, Germany).

### 2.3 LC-MS Analysis

Analysis was performed on a Waters Acquity ultraperformance liquid chromatography (UPLC) controlled by MassLynx 4.1, using a reversed phase (RP) an ACQUITY UPLC 1.8 μm 2.1 × 100 mm HSS T3 column (Waters, Manchester, UK), maintained at 30 °C, coupled to a SYNAPT quadrupole time-of-flight mass spectrometry (QTOF–MS) (Waters, Manchester, UK). Briefly, the chromatographic elution used water/formic acid (99.9:0.1, v/v) as mobile phase A and methanol/formic acid (99.9:0.1, v/v) as mobile phase B, with a 60 min RP run: 100% A was maintained for 6 min, then mobile phase B was increased linearly to 100% over 56 min and held until the end of the run. The flow rate was 0.3 mL/min and the injection volume was 5 μL. All MS instrumental parameters have been previously published.^23^ The samples were fully randomized within each analytical batch which corresponded to the year of collection. QCs were injected every six real samples to monitor signal stability and analytical drift, and a system-suitability standard mixture (STDmix) was injected after every 12 real samples, placed immediately after the corresponding QC injection. In the beginning, the end and during the sequence (about once per day) a blank (solvent) sample was also injected.

### 2.4 Data Processing and Analysis

Data was processed using both a pseudotargeted and an untargeted approach. For the first, TargetLynx tool from MassLynx 4.1 software was used for the pseudotargeted integration of the annotated grape-relevant metabolites. The parameters of the integration were set to 0.08 Da for the chromatogram mass window, 0.2 min for the (retention time—rt) window, and smoothing iterations of 1 and width of 2. These pseudotargeted data were used to interpret the output of the unsupervised analyses obtained from the untargeted feature table.

For the processing following an untargeted approach, raw LC-MS data files were converted to the open mzML format using the MSCconvert tool of ProteoWizard. All subsequent data processing was performed in R using the “xcms” package.^26^ Positive and negative ionization modes (ESI– and ESI+) were processed separately. Spectra were filtered to retain only those acquired within the first 47 min of the chromatographic runs. Peak detection was carried out using the centWave algorithm with the following parameters: ppm = 20; peak width = 6–48 s; signal-to-noise threshold = 5; prefilter = c­(5, 10); noise = 0; mzdiff = 0.001; integrate = 2. Detected chromatographic peaks were subsequently refined using the “refineChromPeaks” function to merge adjacent peaks (expandRt = 9 s; expandMz = 0.001; minProp = 0.75). Rt correction was performed using the “adjustRtime” function with the peak group method. Alignment was based on QC samples, and the following parameters were applied: minFraction = 1; span = 0.4, subsetAdjust = “average”. Chromatographic peaks were grouped across samples using the peak density method, with sample groups defined by grape variety. Grouping parameters were: minFraction = 0.75; binSize = 0.02; bandwidth (bw) = 30. Finally, missing peak intensities were filled in using the “fillChromPeaks” function with the default settings from “ChromPeakAreaParam”, generating the final feature intensity matrix for downstream statistical analysis.

Missing values were assumed to represent signals below the detection limit and were imputed feature-wise using random values between zero and the minimum observed intensity of the corresponding feature. Following imputation, intensity values were log10-transformed to compensate for the expected non-normality distribution of metabolomics data. Batch correction was performed by mean-centering the individual features within each analytical batch (corresponding to sampling year), using QC and grape samples. Feature filtering was based on a comparison of relative standard deviation (RSD, %) between QC and grape samples. RSD values were calculated on back-transformed normalized intensities in linear space. Features were retained when biological variability exceeded analytical variability.

Principal component analysis (PCA) was conducted to get an overview of the main sources of variations within raw and normalized data sets. Log10-transformed and batch-normalized intensities were standardized by mean-centering and scaling to unit variance (autoscaling) across features prior to PCA. The analysis was performed applying the “prcomp” function in R on all detected features when using raw data and on filtered features when using normalized data set.

Uniform Manifold Approximation and Projection (UMAP) was implemented using the “uwot” R package. The primary optimized parameter was “n_neighbors”, whereas all other parameters were left at default settings. To quantitatively assess embedding structure, density-based clustering was performed using Hierarchical Density-Based Spatial Clustering of Applications with Noise (HDBSCAN), as implemented in the “dbscan” R package. Because HDBSCAN assigns ambiguous samples to cluster 0, these samples were reassigned to the cluster of their nearest neighbor in UMAP space to allow consistent computation of silhouette scores across models. Clustering performance was evaluated using the mean silhouette score, which quantifies cluster separation and compactness.

To evaluate the robustness of the data representation, multiple UMAP models were generated across a broad range of “n_neighbors” values and random seeds (100 repetitions per tested value). For each UMAP projection, HDBSCAN was applied across a range of “minPts” values, which determines the minimum number of samples required to define a dense region, thereby influencing the minimum cluster size and the robustness of cluster detection. Finally, only models producing a biologically interpretable number of clusters were considered, excluding trivial or overfragmented solutions. For further details concerning UMAP analysis see Supporting Information (UMAP–HDBSCAN Optimization and Figures S1–S3). Finally, to assess the consistency of sample clustering, we generated 100 UMAP models by randomly sampling “n_neighbors” and “minPts” within the identified ranges to evaluate the stability of sample assignments. From these iterations, a representative model was selected for downstream analysis and visualization to illustrate the clustering behavior and spatial distribution.

### 2.5 Metabolite Annotation

The annotation was performed using the MetaboAnnotation R package.^27^ Candidate compounds were compiled from an internal database containing more than 500 metabolites from different chemical classes^28^ and the theoretical *m*/*z* values of expected adducts were calculated. Feature annotation was achieved by matching theoretical database ions with detected features from the processed data sets using an *m*/*z* tolerance of 0.01 Da and a rt tolerance of 0.5 min, taking into account the ionization mode. Rt used for matching corresponded to those previously obtained in the TargetLynx-based approach, together with those of standards analyzed under the same conditions. Annotation levels were assigned according to Sumner et al.^29^ The complete list of metabolites annotated in the present grapevine collection, including compound name, metabolite class, RT, molecular formula, ionization mode, observed *m*/*z* values, detected adducts/ions, database identifiers where available, and annotation level, is provided in Table S2. All data and metadata were managed in accordance with the Findable, Accessible, Interoperable, and Reusable (FAIR) guidelines.^30^

### 2.6 Data Availability

The code for the full data processing and analyses is provided in R Markdown files in the GitHub repository https://github.com/mar-garcia/grape_metabolome. LC-MS raw data has been deposited in MetaboLights (https://www.ebi.ac.uk/metabolights/MTBLS14377).^31^

## 3 Results and Discussion

The comprehensive GM project analyzed 462 berry samples from 126 grapevine accessions across four consecutive vintages, representing extensive taxonomic, genetic and phenotypic diversity. As shown in Table S1, the final panel included 39 accessions of the Mediterranean ecogeographical group, 13 of the Muscat-flavored group, and 16 of the Central European group, as well as 7 *V. vinifera* cultivars classified within the sylvestris group. To capture intra-*vinifera* genetic stratification, the panel additionally included 30 accessions showing partial membership to multiple STRUCTURE clusters, as well as six accessions assigned to the admixed group (ADM). Finally, accessions with American wild species background were also included as an outgroup together with relevant accessions not analyzed by Emanuelli et al.^20^

To manage the vast scale and complexity of this biodiversity panel, a rigorous analytical workflow was implemented. The metabolome was extracted using an optimized three-solvent biphasic system (described in Section 2.2) to maximize feature yield, and subsequent RP–LC–MS measurements across both ESI+ and ESI– modes required thorough quality control and batch correction to mitigate technical bias from long-sequence runs, which extended over approximately 2 months. As shown in Figure 1, 38% of the injections were dedicated to quality control and data validation to monitor instrumental stability and data quality. This complex design ensured that the final data set provided a robust reference for interpreting metabolomic variability within the grapevine germplasm.

Before interpreting the biological structure of the data set, we first assessed the effect of normalization and batch correction by PCA (Figures 2, Figure S4A–F). The score plots of the non-normalized data set, particularly when colored by analytical batch (Figure S4C,D), suggested that a discernible batch effect was present, especially in ESI+, where samples from the 2007 vintage initially clustered apart from subsequent years. Following normalization, this batch effect was successfully corrected, resulting in a more balanced distribution of samples across batches (Figure S4E,F). The plot of the normalized data provides a clear overview of the main sources of biological variability in the data sets. In both ionization modes, the distribution of samples remained highly consistent, separating into three broad chemotypes corresponding to *V. vinifera* white, *V. vinifera* red, and non-vinifera accessions, with pink and hybrid occupying intermediate positions, reflecting their transitional biochemical profiles. This large-scale structure is consistent with the markedly different chromatographic fingerprints among these groups. For instance, red-skinned accessions are characterized by pigmentation-related phenolics compared with white-skinned accessions, whereas non-vinifera accessions display distinct peak patterns and intensity distributions across the chromatogram (Figure S5).

**Figure 2:**
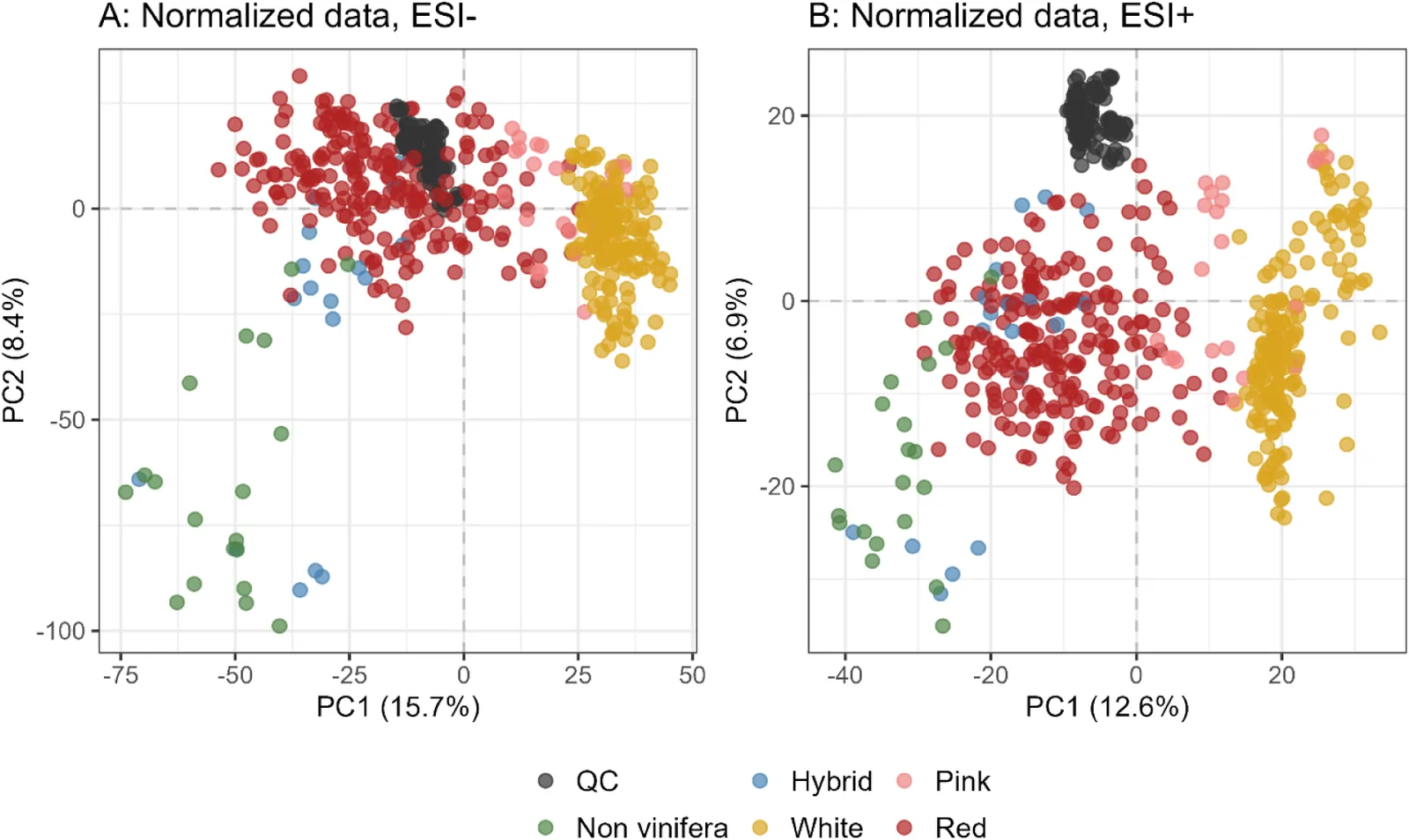
PCA score plots of the untargeted RP–LC–MS data set acquired in ESI– (A) and ESI+ (B) after normalization and batch correction. Samples are colored by taxonomic classification for non-vinifera and hybrid groups and by berry color for *V. vinifera* samples. Corresponding PCA score plots of the raw data are shown in Figure S4.

### 3.1 UMAP-Based Metabolome Map of Grape Biodiversity

Due to its linear nature, PCA is sensitive only to the global structure of the data set, which could mask the local similarity and clustering among the compositional profiles of the different accessions. This limitation is particularly relevant in the GM data set since LC-MS features are expected to show variable patterns across samples which arise not only from direct correlations but also from pathway-linked covariation and cultivar-specific metabolic modules. To better highlight these patterns, we chose to apply UMAP to obtain an interpretable two-dimensional map (embedding) of the GM data set. Unlike PCA (a linear projection maximizing variance), UMAP preserves local manifold structures in nonlinear, high-dimensional data while still maintaining a meaningful global arrangement of major chemotypes. In practice, this improves the visualization of compact subclusters (closely related cultivars/chemotypes) and supports interactive exploration of large biodiversity panels.^32,33^ It is important to highlight that due to the nonlinearity of UMAP, its dimensions cannot be interpreted in terms of “loadings” of the initial variables in a straightforward way. Accordingly, in the following sections, we use an indirect approach based on the distribution of key metabolites from the pseudo-targeted data set; overall, the map should be interpreted as a representation of similarity among multivariate metabolomic profiles.

As described in the Materials and Methods and Supporting Information (UMAP–HDBSCAN Optimization section), a systematic exploration of clustering behavior was performed by generating multiple UMAP projections across a wide range of “n_neighbors” and “minPts” values. The resulting projections produced 2–3 clusters per model. Considering our initial hypotheses, subsequent analyses focused on models with 3 clusters, as these were expected to provide the most informative sample groupings. As anticipated, individual samples were not always assigned to the same cluster across models; these variable assignments, corresponding to samples located at cluster boundaries or between adjacent clusters, are indicated in Figure 3, which presents the UMAP “metabolome map”. As expected, the global structure preserves a clear macro-structure consistent with grape chemotypes, as already observed by PCA (Figure 2). At the largest scale, samples are organized into (i) a *V. vinifera* white region, (ii) a *V. vinifera* red region, and (iii) an “Aromatic” accessions region (Table S1). The isolation of the “Aromatic” accessions as a third group, distinct from the *V. vinifera* red and white clusters, reflects a significant divergence in their metabolomes.

**Figure 3:**
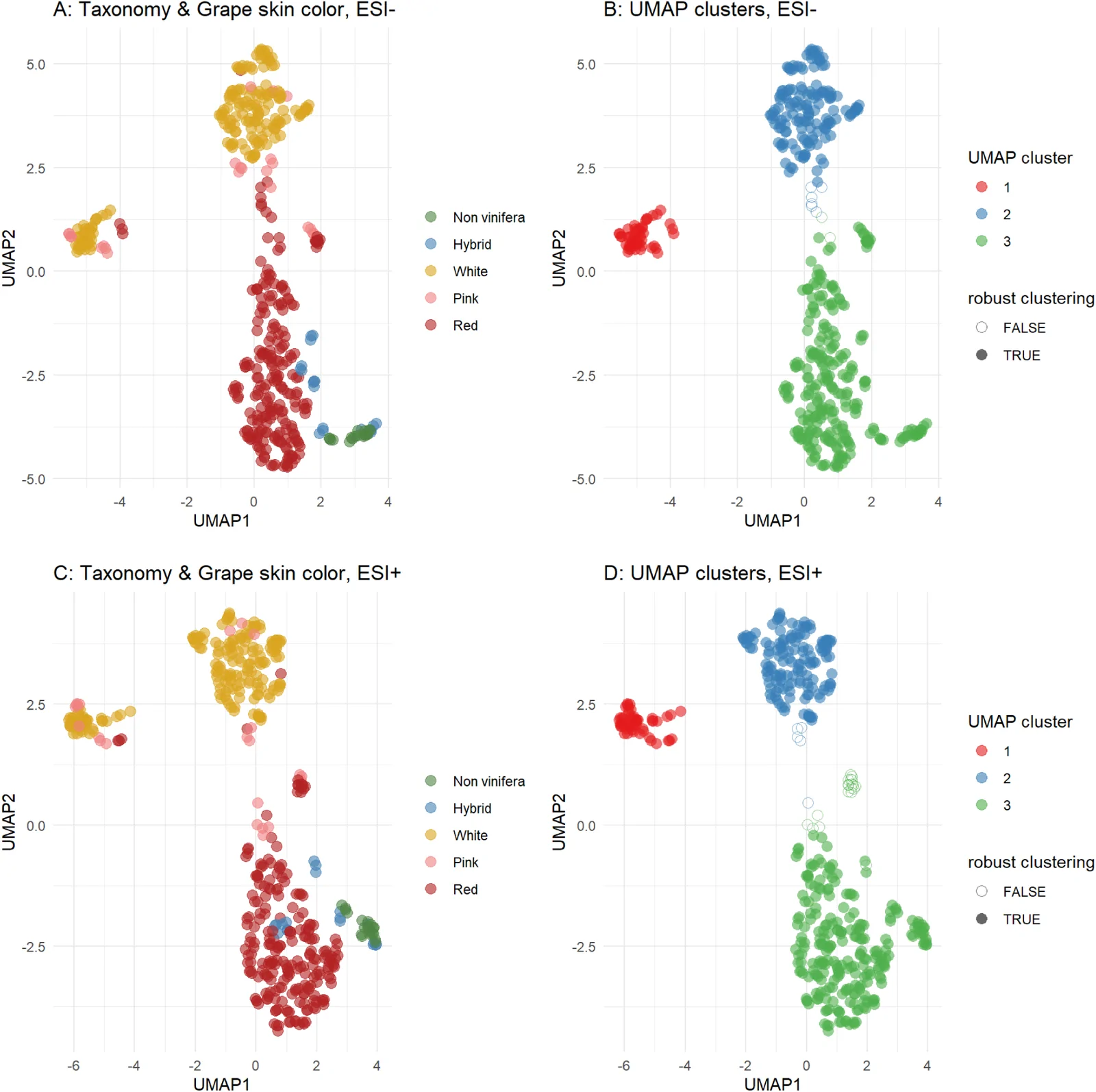
Representative UMAP plots of the untargeted RP–LC–MS data set acquired in ESI– (A-B) and ESI+ (C–D), colored according to taxonomic classification for non-vinifera and hybrid groups, while *Vitis vinifera* samples are colored according to grape skin color (A, C) and UMAP clustering (B, D). Samples indicated with empty circles (B, D) represent those that were not consistently assigned to the same UMAP group.

While the PCA separates the non-vinifera and some hybrid samples along the first two principal components, these samples do not form an entirely independent cluster in the UMAP projection. Instead, they remain associated with the periphery of the *V. vinifera* red cluster (cluster 3), suggesting that despite their taxonomic differences, they share a foundational core metabolic signature with the red cultivars. In contrast, the “Aromatic” group forms a highly resolved, independent cluster, indicating that its chemical signature prevails over the common metabolic background shared by most *V. vinifera* cultivars. Thus, the UMAP suggests that this profile represents a distinct chemotype rather than a marginal variation within the standard GM, indicating that aromatic phenotypes are major drivers of metabolic diversity in this collection. The separation of “Aromatic” samples in the UMAP space is supported by their LC–MS profiles (Figures S5–S6). Representative chromatograms of Gewürztraminer, Moscato Ottonel, and Moscato Rosa show a diagnostic chromatographic region enriched in coeluting signals that are consistently observed across these aromatic samples (Figure S6). This region is consistent with the presence of glycosylated terpene/aroma precursors, which are known to be abundant in Muscat/Gewürztraminer-type cultivars and can dominate cultivar discrimination even across different berry colors (the specific annotated features used to represent this block are listed in Table S2). The shared occurrence and intensity of this “aroma-precursor block” provides a direct chemical explanation for why these accessions cluster together in the UMAP map independently of skin color. Notably, the consistent detection of this diagnostic region in the present untargeted data set prompted the design of a dedicated LC–MS experiment from our group, specifically optimized for the structural characterization of glycosylated aroma precursors.^34^ The interpretation of this region is also supported by independent LC–MS/MS studies on monoterpene glycosides and related aroma precursors in wine grapes.^35^

As expected, the embedding plot also shows additional grouping among the samples, as indicated in Figure 4. The subgroup assignments (e.g., Aromatic, Pinot, Bordeaux, Lambrusco, Rhône, Bianchetta, Terlaner and Riesling) are reported in Table S1 and are discussed below. UMAP also revealed structure among the nonvinifera accessions. Direct interspecific hybrids between American *Vitis* species and *vinifera* cultivars would be expected to cluster closer to the wild group; however, our results indicate that those selected as rootstocks colocalize with American accessions (Millardet et Grasset 41B and Couderc 93-5), whereas those selected for fruit production (Isabella) shift toward the *V. vinifera* accessions. Thus, our data suggests a gradient from wild-like to vinifera-like metabolomic space: the early rootstock hybrids described above, colocalized with the wild accessions, whereas Isabella shifted toward the *V. vinifera* region close to Plantet, an advanced interspecific hybrid characterized by a higher *V. vinifera* genomic contribution given by its pedigree. Finally, Pinot noir × Merzling (P × N), a later-generation hybrid resulting from successive breeding cycles, was nearly superimposed on vinifera samples (Figure 7). Another interesting feature of the projection is the presence of a compact “Pinot” subcluster grouping Pinot noir and its somatic color variant Pinot gris. Despite their marked differences in berry pigmentation, which are associated with altered anthocyanin accumulation, the two accessions remained metabolically close, suggesting that these pigment-related changes have a limited impact on the overall metabolomic fingerprint or are balanced by variation in other metabolite classes. Within the vinifera regions, additional substructure matched known cultivar “families” or shared enological lineages (Table S1), including the “Bordeaux” (Cabernet Sauvignon/Cabernet Franc/Merlot), Lambrusco and Bianchetta subgroup, and smaller white skinned subgroups (e.g., Rhône; Xarello–Vermentino), highlighting that the data set captures both broad chemotaxonomic separation and finer cultivar-level relationships.

**Figure 4:**
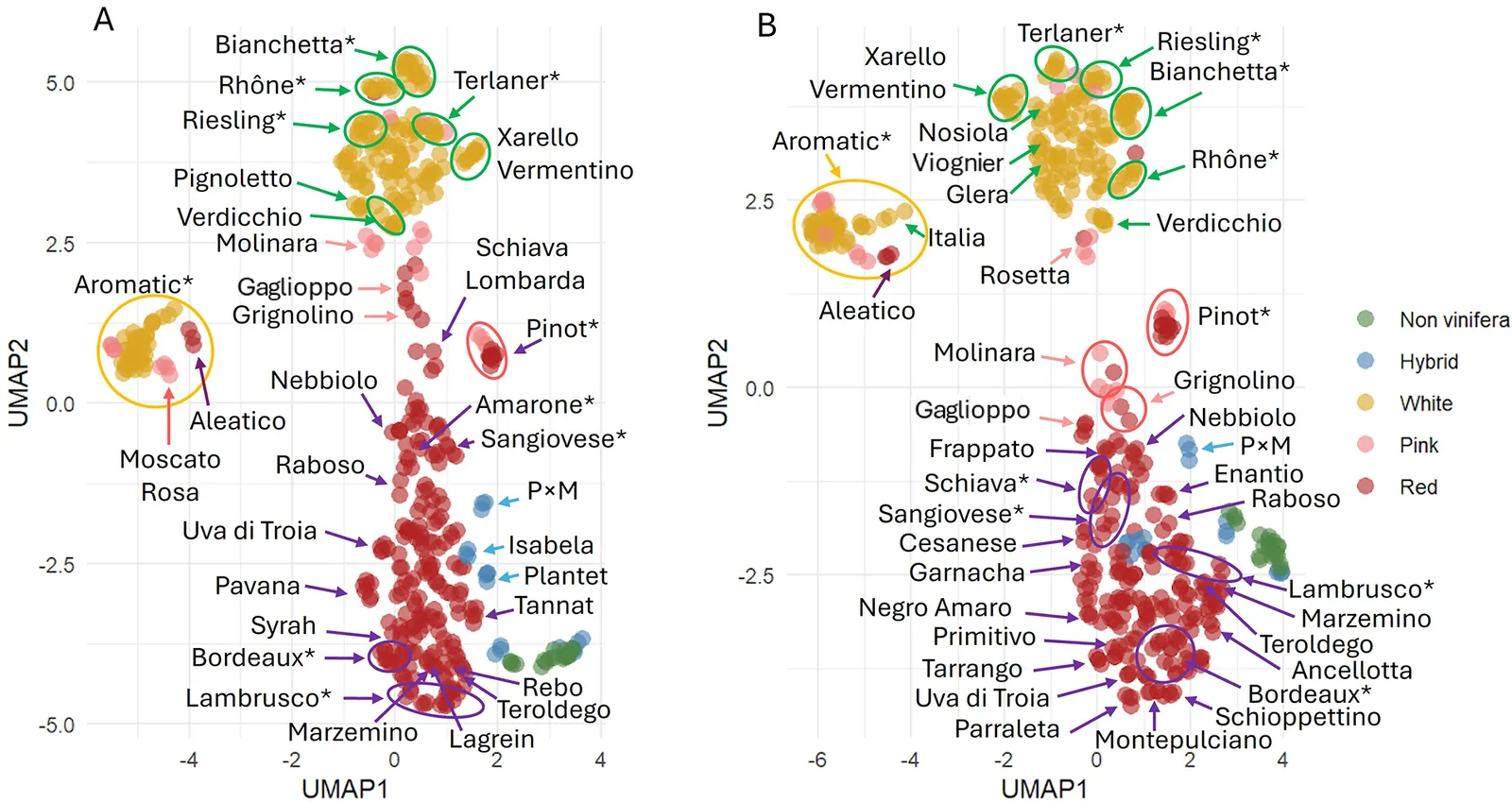
UMAP-based “metabolome map” of the grape biodiversity panel built from the untargeted RP–LC–MS data set acquired in ESI– (A) and ESI+ (B). Each point represents one accession/sample; colors indicate taxonomic classification for nonvinifera and hybrid groups or grape skin color (white, pink/gray, red) for *Vitis vinifera* samples. Ellipses and labels highlight the main chemotype regions and representative subclusters, including nonvinifera, hybrids, and selected vinifera subgroups* within red and white samples (e.g., Pinot, Aromatic, Lambrusco, Bordeaux, Xarello–Vermentino, Rhône, Bianchetta, Terlaner, Riesling – see Table S1). Arrows indicate the approximate position of representative cultivars.

To provide a more direct chemical interpretation of the UMAP-based “metabolome map” and link the unsupervised clustering to specific biochemical drivers, we leveraged compound annotation. This was achieved by projecting the relative abundance of representative key metabolites from the pseudotargeted processing approach onto the UMAP coordinates to visualize the chemical basis of the sample organization.

### 3.2 Chemical Interpretation of the Metabolome Map

Feature annotation was based on our internal standard injections database, which includes several hundred of metabolites from different chemical groups,^28,36^ together with evidence from previous *Vitis*-related studies,^11,14,37–39^ The results are summarized in Table S2, which contains 116 level-1 (identification with standards), 26 level-2 and 30 level-3 annotations. Most annotated metabolites belong to the group of phenols/polyphenols covering the biosynthetic pathways of a) glucogalloyl derivatives, b) cinnamates, c) stilbenoids, and d) flavonoids, including anthocyanins, flavanols, flavones and flavanols. Given the importance of the phenolic profile in the classification of the grape cultivars, this provides an important tool for the wine science community.

Figure 5 highlights the distribution of different anthocyanin-related variables across the GM map. As expected, the sum of all monoglucosides of anthocyanins showed high abundance in the red region and low abundance in the white region (Figure 5A). At a finer level, acylated anthocyanins showed a more structured distribution within red cultivars (Figure 5B). Anthocyanin 3,5-diglucosides were concentrated in the nonvinifera area and specific hybrids (Figure 5C), supporting their use as markers of nonvinifera contribution. In Figure 5D, samples were colored according to the summed abundance of terpene precursors, highlighting the “Aromatic” group but also elevated levels in accessions outside this cluster, such as Aleatico and Nero, which are also known to display Muscat-like character. Nero is a disease-resistant table-grape cultivar with reported enological potential and an aromatic profile consistent with elevated monoterpenol levels in wine.^40^

**Figure 5:**
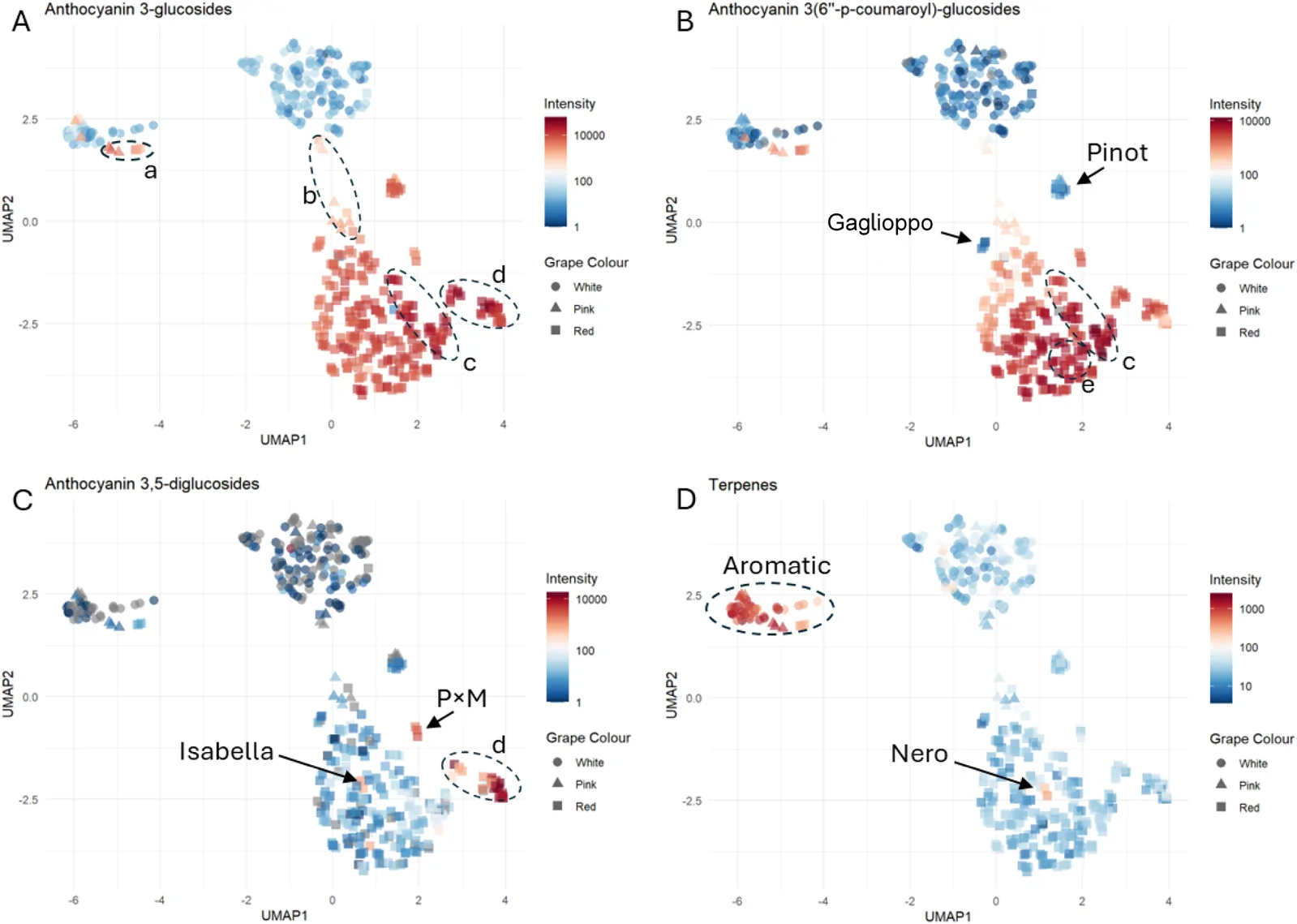
Projection of anthocyanin-related variables onto the UMAP “metabolome map” (ESI+). Each point represents one sample/accession positioned according to its UMAP coordinates (same embedding as in Figure 3), while the color scale reports the relative abundance (peak area) of the indicated anthocyanin group/marker. (A) Sum of anthocyanin 3-glucosides. (B) Sum of acylated anthocyanins, exemplified by anthocyanin 3­(6″-*p*-coumaroyl)-glucosides. (C) Sum of anthocyanin 3,5-diglucosides. (D) Sum of terpenes (Table S2). a: Aleatico and Moscato Rosa, b: pink samples, c: Lambrusco, d: Nonvinifera and Hybrids, e: Bordeaux group, arrows indicate representative samples discussed in the text.

Using the same approach adopted for anthocyanins, flavonol composition patterns were further investigated through ratios reflecting B-ring hydroxylation classes (Figure 6). Both the flavonol monoOH/triOH (Figure 6A) and diOH/triOH (Figure 6B) ratios displayed structured gradients across the UMAP space, highlighting distinctive profiles for specific cultivar groups. These overlays provide a pathway-oriented interpretation of the chemical variation underlying the observed clustering and complement the patterns described above.

**Figure 6:**
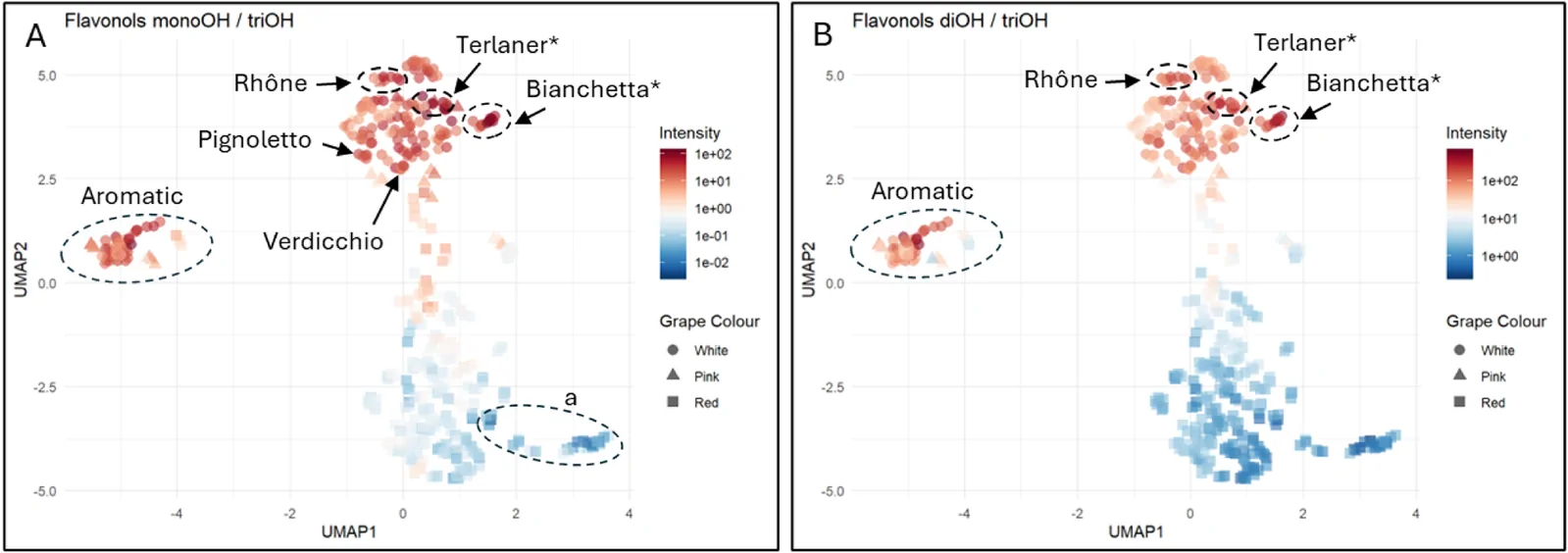
Projection of flavonol hydroxylation-pattern ratios onto the UMAP metabolome map (ESI−). Each point represents one sample positioned according to its UMAP coordinates (same embedding as in Figure 4), and the color scale indicates the relative value of the ratio calculated from the summed peak areas of annotated flavonols belonging to each hydroxylation class, as indicated in the panel titles. (A) Flavonols: monoOH/triOH. (B) Flavonols: diOH/triOH (Table S2). The structured gradients across the map indicate that differences among cultivar/species groups are reflected in flavonol hydroxylation patterns.

To support the interpretation of Figure 6, Figure S7 visualizes representative flavonol glucosides spanning the main B-ring hydroxylation classes (kaempferol-, quercetin-, and myricetin-type – Table S2). The distinct spatial distributions of these markers closely reproduce the gradients observed for the hydroxylation-class ratios, indicating that the overlays in Figure 6 reflect coordinated remodeling of the flavonol pool rather than the behavior of a single outlier feature. This pattern is consistent with previous grape studies showing that white-skinned cultivars are enriched in quercetin- and kaempferol-type flavonols and largely lack myricetin-type derivatives, whereas red cultivars accumulate appreciable proportions of both di- and trihydroxylated flavonols.^41^ Considering biosynthetic pathways, these differences are compatible with genotype-dependent variation in the relative contribution of the flavonoid 3′-hydroxylase (F3′H) and flavonoid 3′,5′-hydroxylase (F3′5′H) branches, which shape the balance between di- and tri-hydroxylated intermediates and, ultimately, the abundance of quercetin- versus myricetin-derived flavonols.^42^

To further examine tannin-related phenolic variation across the grape biodiversity panel, we compared the distribution of summed dimeric procyanidins type B and of monogalloyl-glucose (glucogallin) across accessions (Figure 7 and Table S2). The profiles showed a broad contrast between *V. vinifera* and nonvinifera/hybrid germplasm: *V. vinifera* accessions were generally richer in proanthocyanidins, whereas nonvinifera and hybrid materials displayed relatively higher levels of glucogalloyl derivatives, consistent with the reported enrichment of hydrolyzable-tannin precursors in wild American grapes compared with *V. vinifera*.^43^ Within vinifera, Pinot noir, Pinot gris, Aglianico, Nebbiolo, Lagrein, and Moscato Rosa were among the most procyanidin-rich accessions, while Lambrusco del Casetta and Croatina showed comparatively high levels of both metabolite groups.

**Figure 7:**
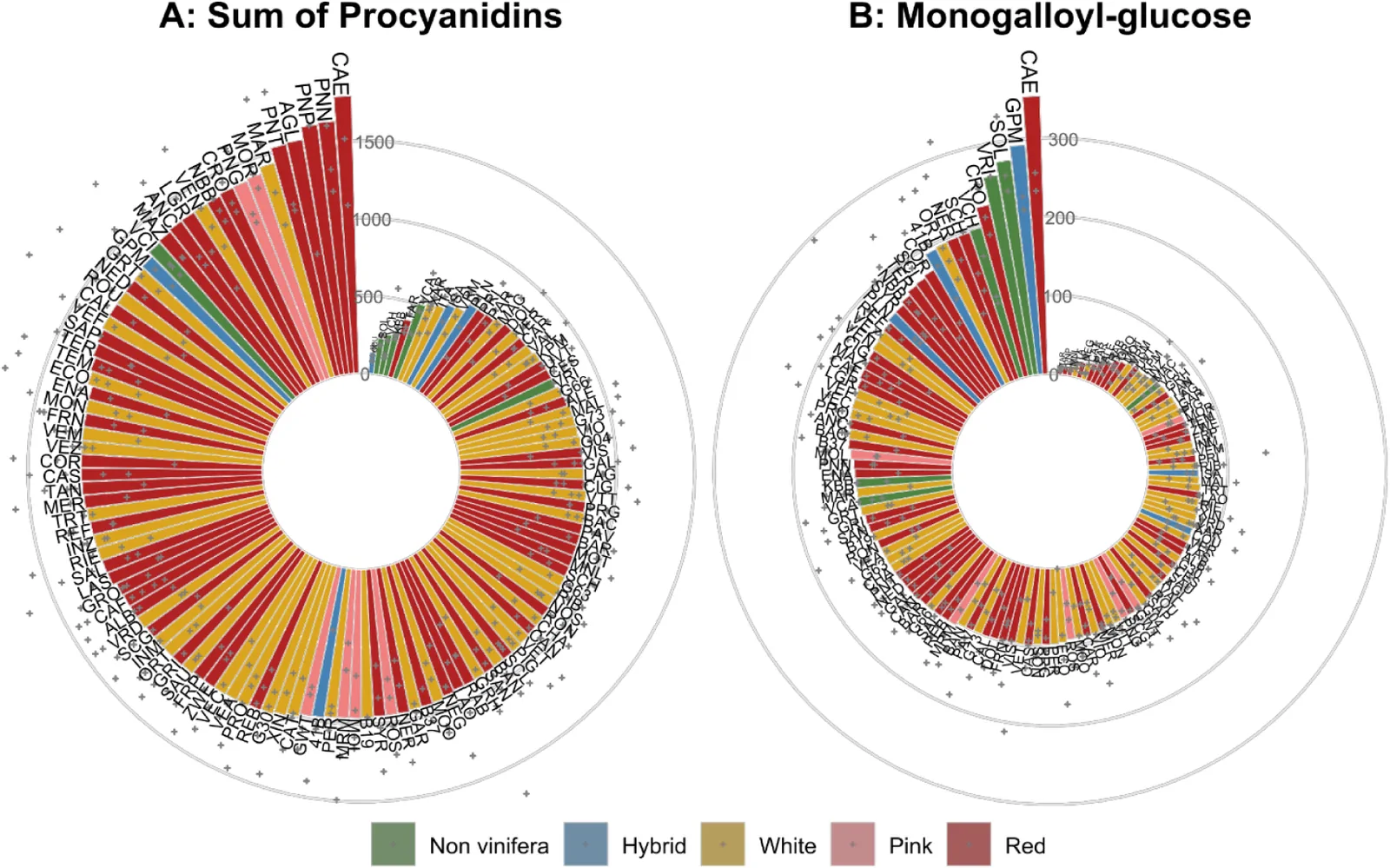
Circular bar plots showing the accession-level abundance of (A) the sum of annotated procyanidins and (B) monogalloyl-glucose across the grape biodiversity panel (Table S2). Bars represent mean values and are colored by germplasm/berry group, and open circles represent individual measured values. Three-letter accession codes are reported in Table S1; among the accessions discussed in the text, these include Pinot noir (PNN, PNP, PNT), Pinot gris (PNG), Aglianico (AGL), Nebbiolo (NBB), Lagrein (LGR), Moscato Rosa (MOR), Lambrusco del Casetta (CAE), and Croatina (CRO).

The accession-level profile of trigalloyl glucose (Figure S8) further highlighted cultivar-specific variation within the glucogalloyl pathway, with Cabernet Franc emerging as a notable outlier. This observation is noteworthy since according to our knowledge is the first time that is reported.

A further cultivar-dependent pattern was observed for indole-3-lactic acid glucoside (Figure S9), an indole derivative first reported in red wine by Fabre et al.^44^ and shown to vary with cultivar/microclimate and to decrease during aging. In our previous studies, the corresponding sulfonated derivative emerged as a novel wine marker promoted by oxygen exposure, suggesting a role in SO_2_ consumption during bottle storage.^37,45^ Varietal differences in this precursor may therefore help explain differences in the fate of SO_2_ during wine evolution.

### 3.3 Broader Significance, Limitations, and Future Perspectives

Previous methodological work supporting the GM resource focused on sample preparation, data processing, annotation, traceability, and reproducibility in large untargeted LC–MS studies. Representative outcomes included extraction protocol optimization,^23^ the development of specific annotation tools,^28,36^ the creation of an end-to-end data processing workflow tailored to untargeted MS-based metabolomics experiments (MetaDB),^46^ and the organization of the data and metadata specific for the grapevine metabolomics experiment according to FAIR principles.^30^ These advances are especially relevant in biodiversity-scale metabolomics, where the challenge is not only to detect features, but also to organize them into a coherent biochemical space for comparison across cultivars, species, and hybrids. Overall, these efforts emphasize that metabolomics is a rigorous analytical framework rather than simply a descriptive label.

In parallel, application studies generated during the project helped demonstrate the value of metabolomics-guided sampling across grape diversity and showed how an expanding knowledge base can support sample selection and hypothesis generation. Examples from our group include the detection of anthocyanins in white grape cultivars,^47^ the mapping of free volatiles and glycosylated aroma precursors able to differentiate cultivars,^34^ and the discrimination of vinifera and nonvinifera germplasm based on both known and unknown metabolomic features.^43^ Taken together, these studies and tools highlight the broader utility of the GM resource. Beyond serving as a reference framework for chemotaxonomy and biodiversity mapping, it can support authenticity and traceability studies by providing a wider metabolomic space than is typically available in small, highly specific data sets. At the same time, it enables hypothesis-driven exploration of links between genotype, pathway architecture, and phenotype, with relevance for molecular breeding and trait discovery. By coupling broad biodiversity coverage with annotation practices aligned with MSI principles and data management compatible with open repositories, the data set becomes a reusable and integrable resource for future reannotation and machine-learning applications.

The chemotaxonomic structure observed here is consistent with established grape biology and previous metabolomic studies. Separation by berry color reflects the major contribution of anthocyanins and related flavonoids and is coherent with the central role of the MYBA locus in pigmentation,^48^ while the grouping of Pinot color variants agrees with their known somatic origin.^49^ The localization of anthocyanin 3,5-diglucosides in nonvinifera and introgressed germplasm supports their use as indicators of nonvinifera background, and the clustering of aromatic cultivars is in line with their terpene-rich metabolome and the involvement of VvDXS in Muscat-like aroma.^50–52^ In addition, the distribution of flavanol and flavonol hydroxylation patterns suggests broader differences in flavonoid pathway architecture across cultivar and species groups. Together, these observations support the value of the data set as a biodiversity-scale reference for grape chemotaxonomy.

This study establishes a comprehensive LC–MS-based grape metabolome resource and a practical framework to explore grapevine biodiversity through chemistry. By combining long-gradient RP–LC–MS profiling in both ESI– and ESI+, rigorous QC monitoring and normalization, and unsupervised multivariate analysis, we generated an interpretable “metabolome map” that consistently resolved the major grape chemotypes while also revealing finer cultivar-level structure and distinctive subgroups such as aromatic cultivars. Overlaying pathway-relevant metabolite families and compositional ratios (e.g., anthocyanin classes and flavonoid hydroxylation patterns) linked clustering patterns to biologically meaningful chemical drivers, providing an intuitive bridge between untargeted fingerprints and specialized metabolism. Overall, the GM data set offers a scalable reference for chemotaxonomy, authenticity/traceability, biodiversity-driven metabolite discovery, and the identification of candidate traits for breeding and precision viticulture and enology. Future work integrating this resource with genetic information and expanded metabolite annotation will further strengthen its value for understanding and leveraging grape metabolic diversity.

## Supplementary Material







## Notes

UMAP–HDBSCAN optimization workflow, together with supplementary figures (Figures S1–S3); tables supporting data processing, chemical interpretation, and accession-level visualization of the grape metabolome data set; Figures S4–S6: provide additional PCA and chromatographic views of the data set, including normalization/batch-correction effects and representative profiles of contrasting and aromatic grape chemotypes; Figure S7: reports additional metabolite comparisons and UMAP projections supporting the interpretation of phenolic variation; Figures S8–S9: show the accession-level distribution of trigalloyl-glucose and indole-3-lactic acid glucoside; sample metadata (Table S1); compound annotation table with associated confidence levels (Table S2) (PDF)
Table S1: samples metadata (XLSX)
Table S2: metabolite annotation information (XLSX)

## #

Biometris, Wageningen University and Research, Droevendaalsesteeg 1, Wageningen, 6708 PB, The Netherlands

## ∇

Laboratory of Analytical Chemistry, School of Chemistry, Aristotle University of Thessaloniki, Thessaloniki, 54124, Greece.

## ○

Center Agriculture Food and Environment (C3A), University of Trento, San Michele all’Adige, 38098, Italy.

## ◆

Center for Food Metabolomics and Bioactives Development (CSMETAB), Trento, 38098, Italy.
