# Supplementary material for: A Metabolome Atlas of Grape Biodiversity
Source: J Agric Food Chem. 2026 Aug 25;74(35):28014–25. doi: 10.1021/acs.jafc.6c04648 (PMC13573557; doi:10.1021/acs.jafc.6c04648)
Supplement: Supplementary file 1 [file jf6c04648_si_001.pdf]

Supporting Information

A Metabolome Atlas of Grape Biodiversity

Panagiotis Arapitsas [a*], Pietro Franceschi [a], Mar Garcia-Aloy [a], Urska Vrhovsek [a], Luca Narduzzi [a], Domenico Masuero [a], Marco Stefanini [a], Paola Bettinelli [a], Maria Stella Grando [a%], Francesco Emanuelli [a], Ron Wehrens [a#], Georgios Theodoridis [a$] and Fulvio Mattivi [a&*]

Research and Innovation Centre, Fondazione Edmund Mach, San Michele all'Adige, 38098, Italy

*Corresponding authors

Panagiotis Arapitsas e-mail: panagiotis.arapitsas@fmach.it

Fulvio Mattivi e-mail: fulvio.mattivi@csmetab.com

UMAP–HDBSCAN Optimization

To determine the optimal ranges for the key parameters of the UMAP-HDBSCAN pipeline and ensure a robust biological representation of the data, the following systematic optimization workflow was employed:

1. Iterative model generation: multiple UMAP models were generated by sweeping the "n_neighbors" parameter from 2 up to the total number of samples. To account for non-deterministic embedding, 100 independent UMAP runs were performed for each tested "n_neighbors" value.
2. Sample clustering: HDBSCAN clustering was applied to each generated UMAP by varying the "minPts" parameter (minimum cluster size) from 2 up to half the total number of samples. Clustering performance was evaluated using the mean silhouette score, which quantifies cluster separation and compactness.
3. Cluster-based model selection: to identify biologically interpretable groupings, models yielding an uninformative number of clusters (fewer than 3 or more than 10) were excluded. Within this subset, the retained models spanned the full range of “n_neighbors” values, while “minPts” values were limited to the ranges 4–65 (ESI-) and 4–70 (ESI+).

4. Silhouette score analysis: silhouette scores for the filtered models (3-10 clusters) are presented as a function of "n_neighbors" and "minPts" for both ionization modes in **Figure S1**. A bimodal distribution in silhouette scores was clearly visible at “n_neighbors” ≥20 for ESI- and ≥25 for ESI+.

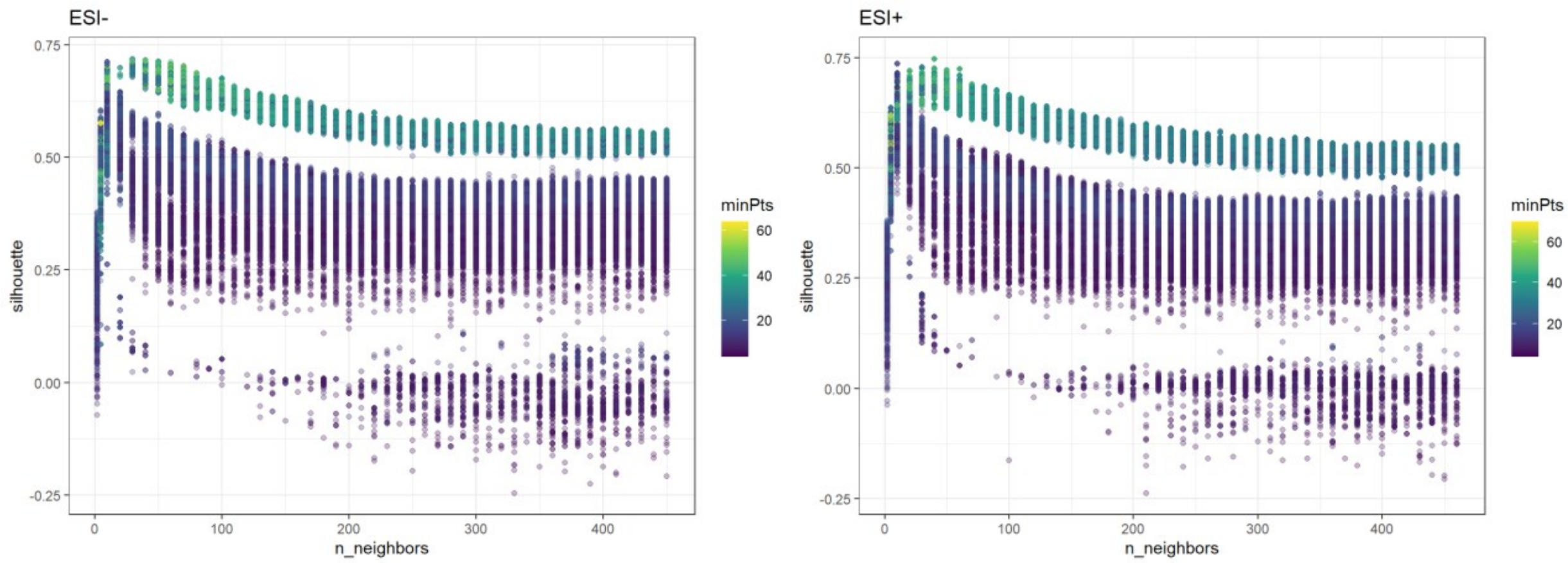

Figure S1. Distribution of silhouette scores according to "n_neighbors" parameter. Each point represents an independent UMAP-HDBSCAN model and is colored according to the “minPts” value used for the corresponding model. Only models yielding 3–10 clusters are included.

The upper component of the bimodal distribution was mainly characterized by “minPts” ≥ 21 in ESI- and “minPts” ≥ 22 in ESI+ (**Fig. S2**).

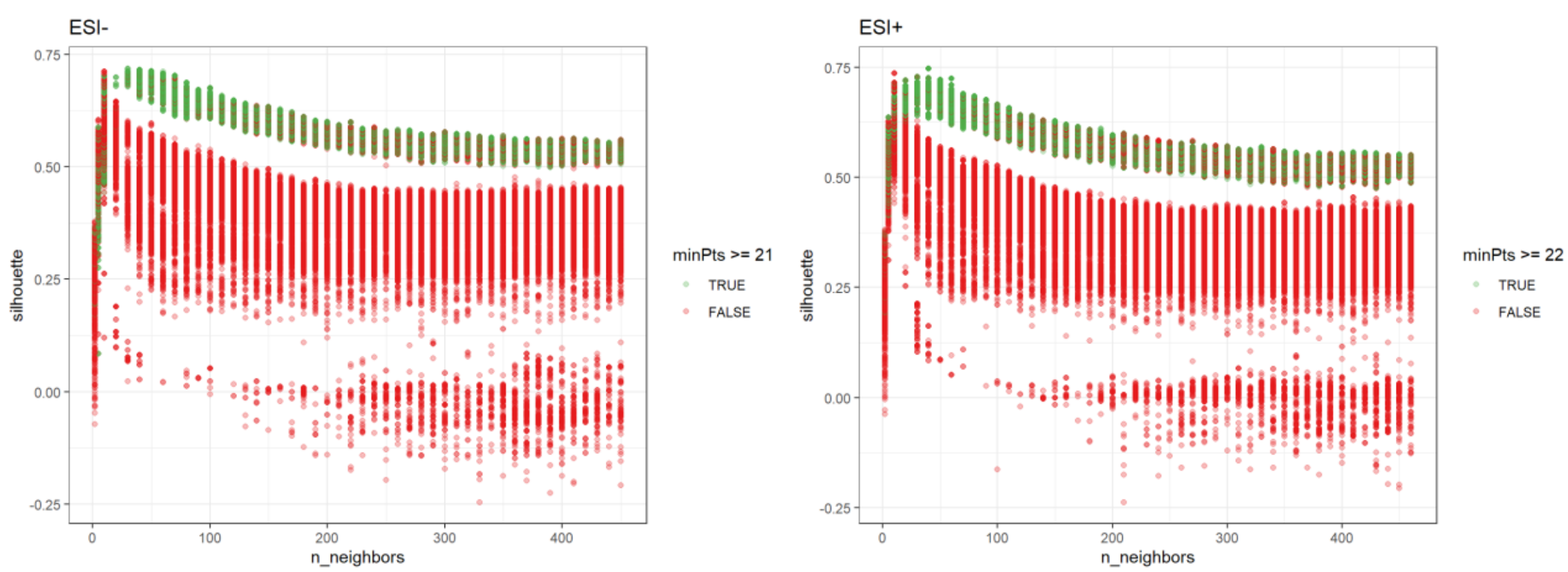

Figure S2. Distribution of silhouette scores according to "n_neighbors" parameter. Each point represents an independent UMAP-HDBSCAN model. Only models yielding 3–10 clusters are included. Color indicates if that model was built with a “minPts” equal or higher to 21 (ESI-) or 22 (ESI+).

5. <u>Final parameter selection:</u> as indicated in **Figure S3** the most stable and highest silhouette scores were obtained within the following ranges, which were used for all downstream analyses:
    - ESI-: "n_neighbors" 27-44 and "minPts" 21-50.
    - ESI+: "n_neighbors" 23-46 and "minPts" 22-50.

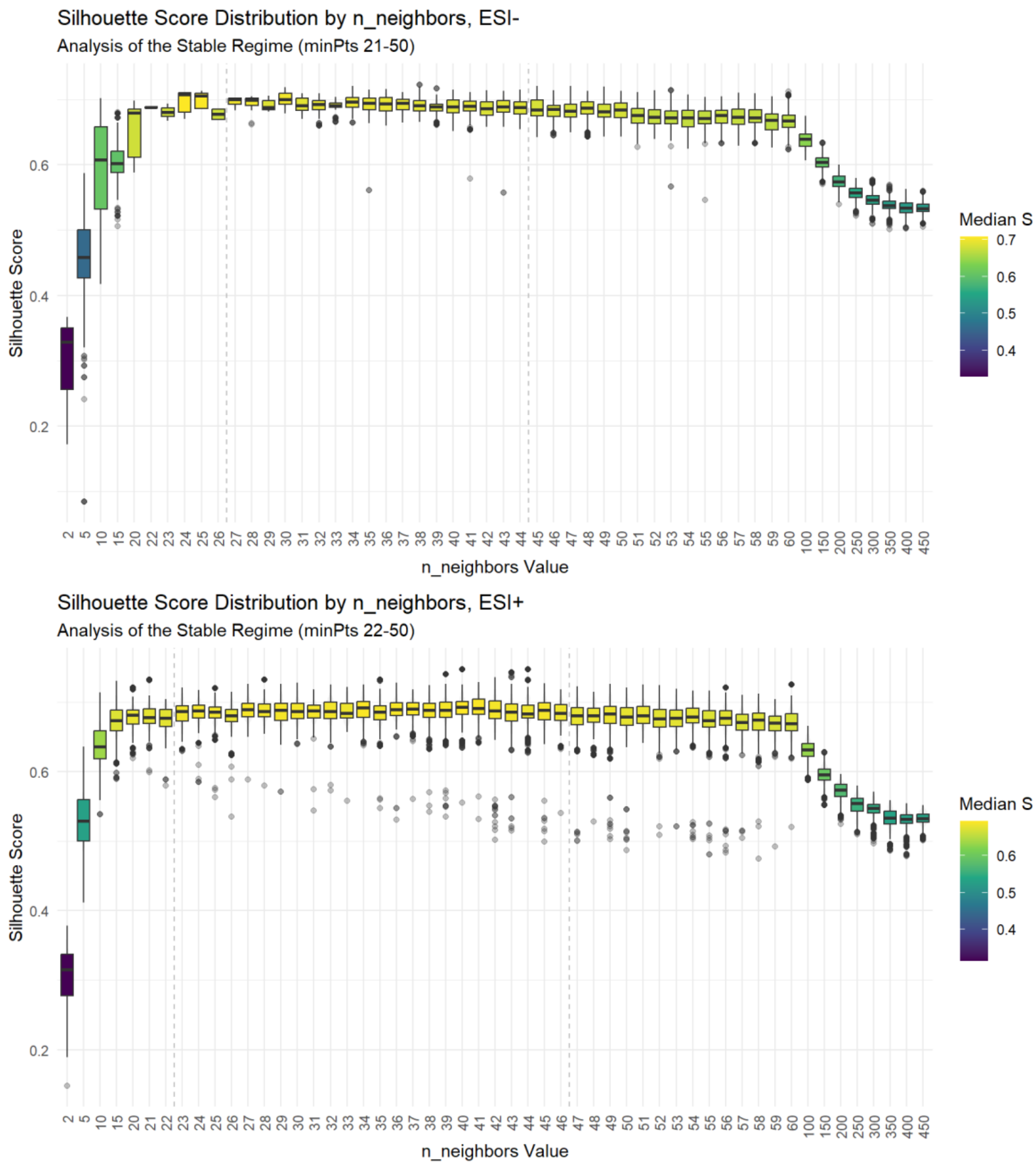

Figure S3. Distribution of silhouette scores according to "n_neighbors" parameter. Each boxplot represents the distribution of silhouette score for models yielding 3-10 clusters within the “minPts” range indicated in the corresponding title. Boxplots are colored by the median silhouette score of retained models within each “n_neighbors” value, while vertical dashed lines indicate the final selected thresholds for “n_neighbors” parameter.

Other Supplementary Figures

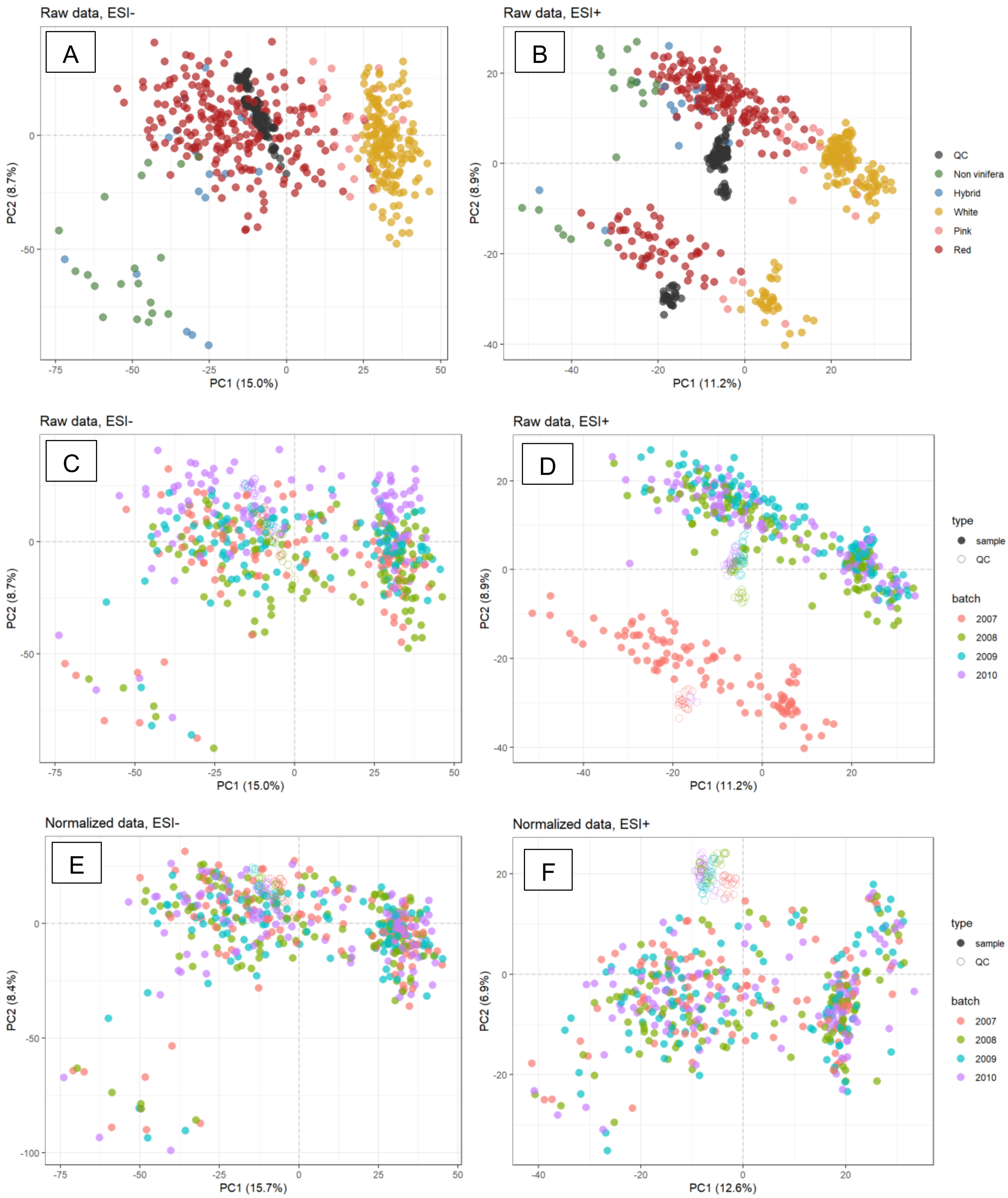

Figure S4. PCA score plots of the untargeted RP-LC-MS dataset acquired in ESI- (left) and ESI+ (right). Samples are colored by taxonomic classification for non-vinifera and hybrid groups and by grape skin color for *Vitis vinifera* samples (first row); or by analytical batch before and after normalization process (second and third row).

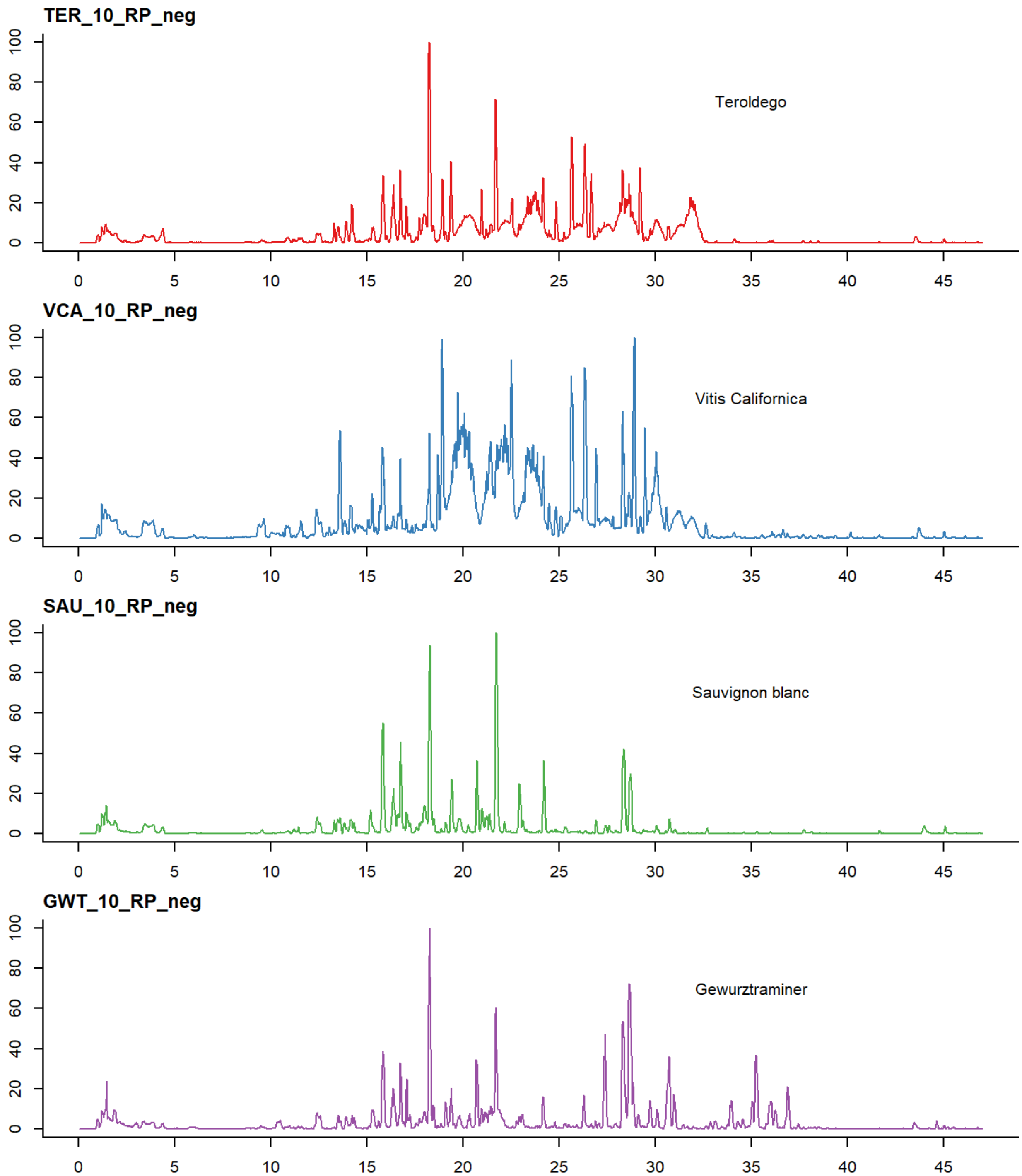

Figure S5. Representative Base Peak Intensity (BPI) chromatograms acquired in ESI− mode for four contrasting grape chemotypes: a red *V. vinifera* cultivar (Teroldego), a white *V. vinifera* cultivar (Sauvignon blanc), an aromatic *V. vinifera* cultivar (Gewürztraminer), and a non-vinifera accession (*Vitis californica*).

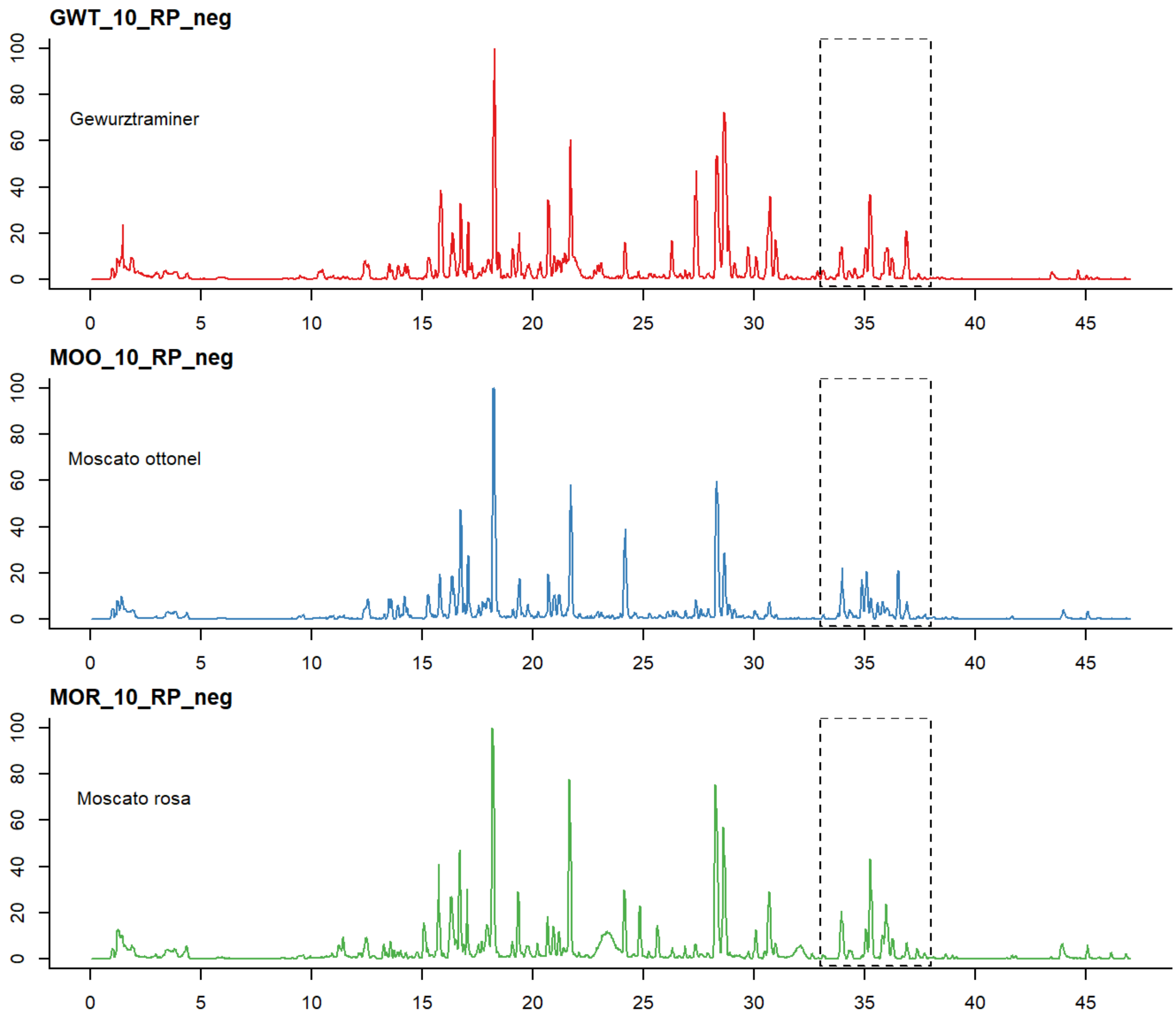

Figure S6. Representative base peak ion chromatograms (BPI) of three aromatic cultivars (Gewürztraminer, Moscato Ottonel, and Moscato Rosa) acquired with the same RP–LC–MS method used for the full dataset. The dashed box highlights a shared late-eluting chromatographic region enriched in these aromatic samples, indicating a characteristic set of signals that likely contributes to their clustering as a distinct "aromatic" subgroup in the UMAP metabolome map.

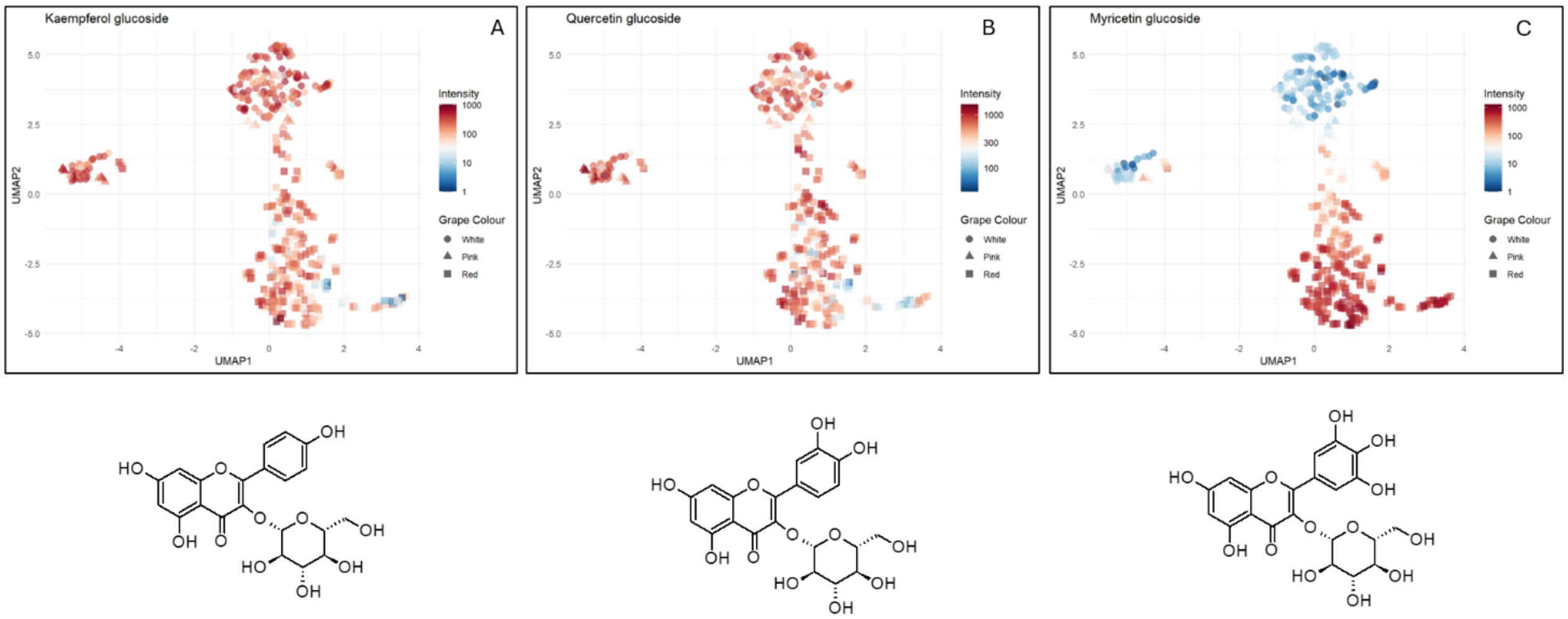

Figure S7. UMAP projection of representative flavonol glucosides supporting the hydroxylation-pattern analysis shown in Fig. 6. Each point represents one sample/accession positioned according to the same UMAP embedding, and the color scale indicates the relative abundance (normalized peak area) of the metabolite. (A) Kaempferol glucoside (representative mono-hydroxylated B-ring flavonol type). (B) Quercetin glucoside (representative di-hydroxylated type). (C) Myricetin glucoside (representative tri-hydroxylated type). The structured spatial distributions provide single-metabolite examples underlying the ratio gradients observed for flavonols in Fig. 6.

trigalloyl glucose

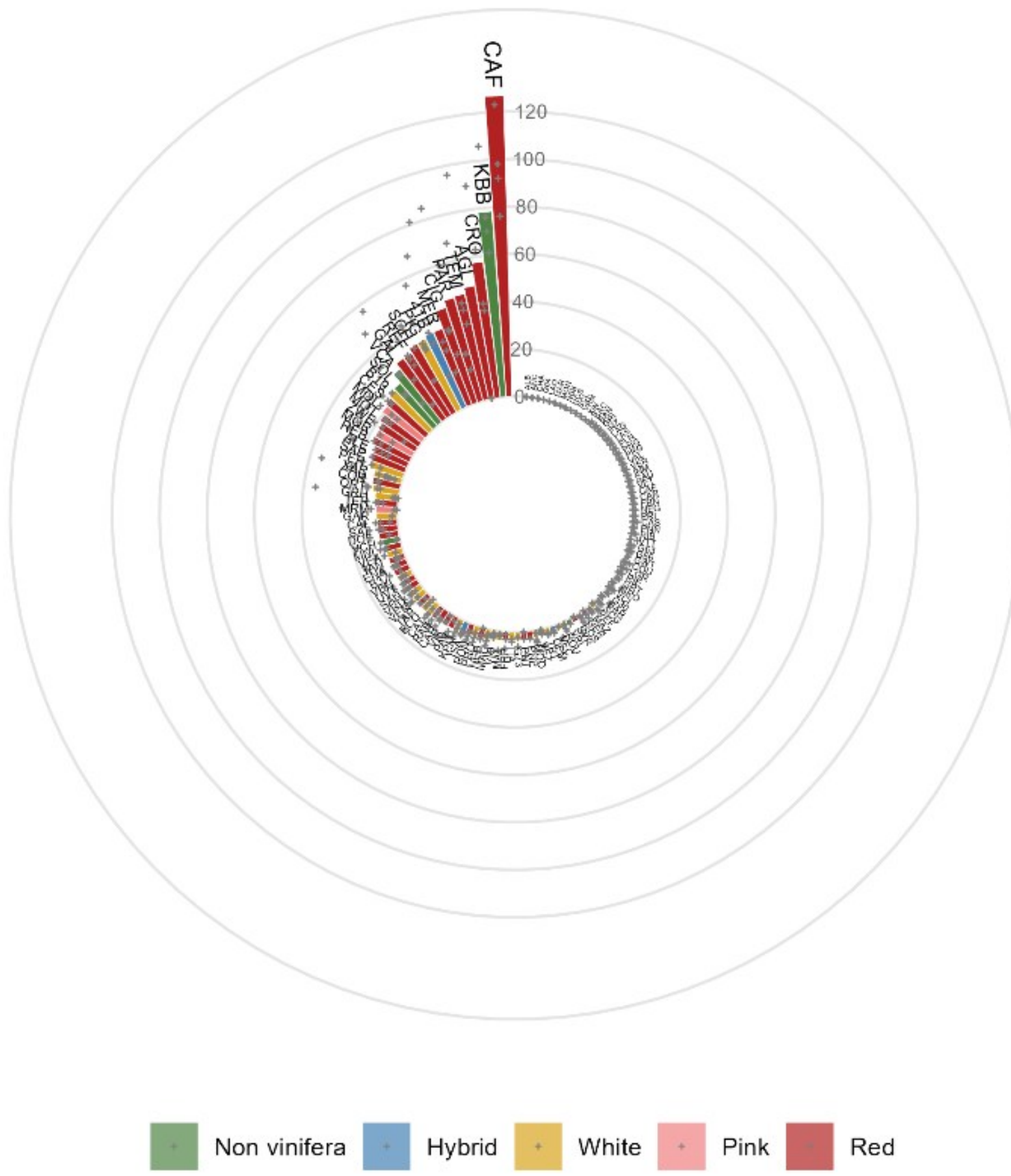

Figure S8. Accession-level distribution of trigalloyl glucose across the grape biodiversity panel. Circular bar plot showing the relative abundance of trigalloyl glucose across grape accessions. Bars represent mean values and are colored according to germplasm/berry group (non-vinifera, hybrid, white, pink, and red), and open circles indicate individual measured values. The plot highlights marked accession-dependent variation within the early stage of gallotannin biosynthesis, with Cabernet Franc (CAF) emerging as a notable outlier among the analyzed materials. The codes explanation can be found in Table S1.

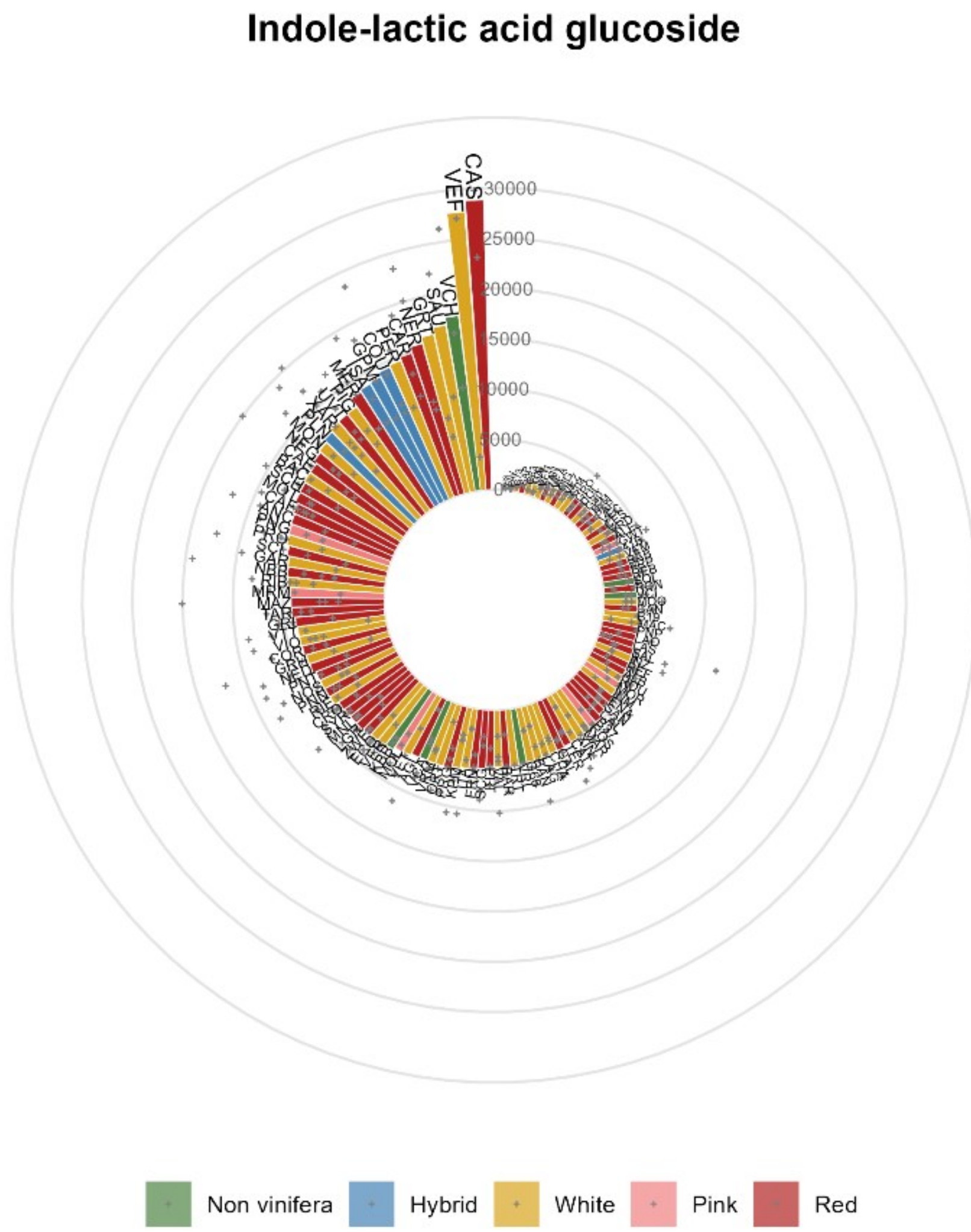

Figure S9. Accession-level distribution of indole 3-lactic acid glucoside across the grape biodiversity panel. Circular bar plot showing the relative abundance of indole 3-lactic acid glucoside across grape accessions. Bars represent mean values and are colored according to germplasm/berry group (non-vinifera, hybrid, white, pink, and red), and open circles indicate individual measured values. The plot highlights marked accession-dependent variation within tryptophan pathway, with the red Cabernet Sauvignon (CAS) and the white Verduzzo friulano (VEF) emerging as a notable outlier among the analyzed materials. The codes explanation can be found in Table S1.
